# Supplementary material for: Brain-wide projection reconstruction of single functionally defined neurons
Source: Nat Commun. 2022 Mar 22;13:1531. doi: 10.1038/s41467-022-29229-0 (PMC8940919; doi:10.1038/s41467-022-29229-0)
Supplement: Supplementary file 1 — Supplementary information [file 41467_2022_29229_MOESM1_ESM.pdf]

Supplementary Information

Brain-wide projection reconstruction of single functionally defined neurons

Wang et al.

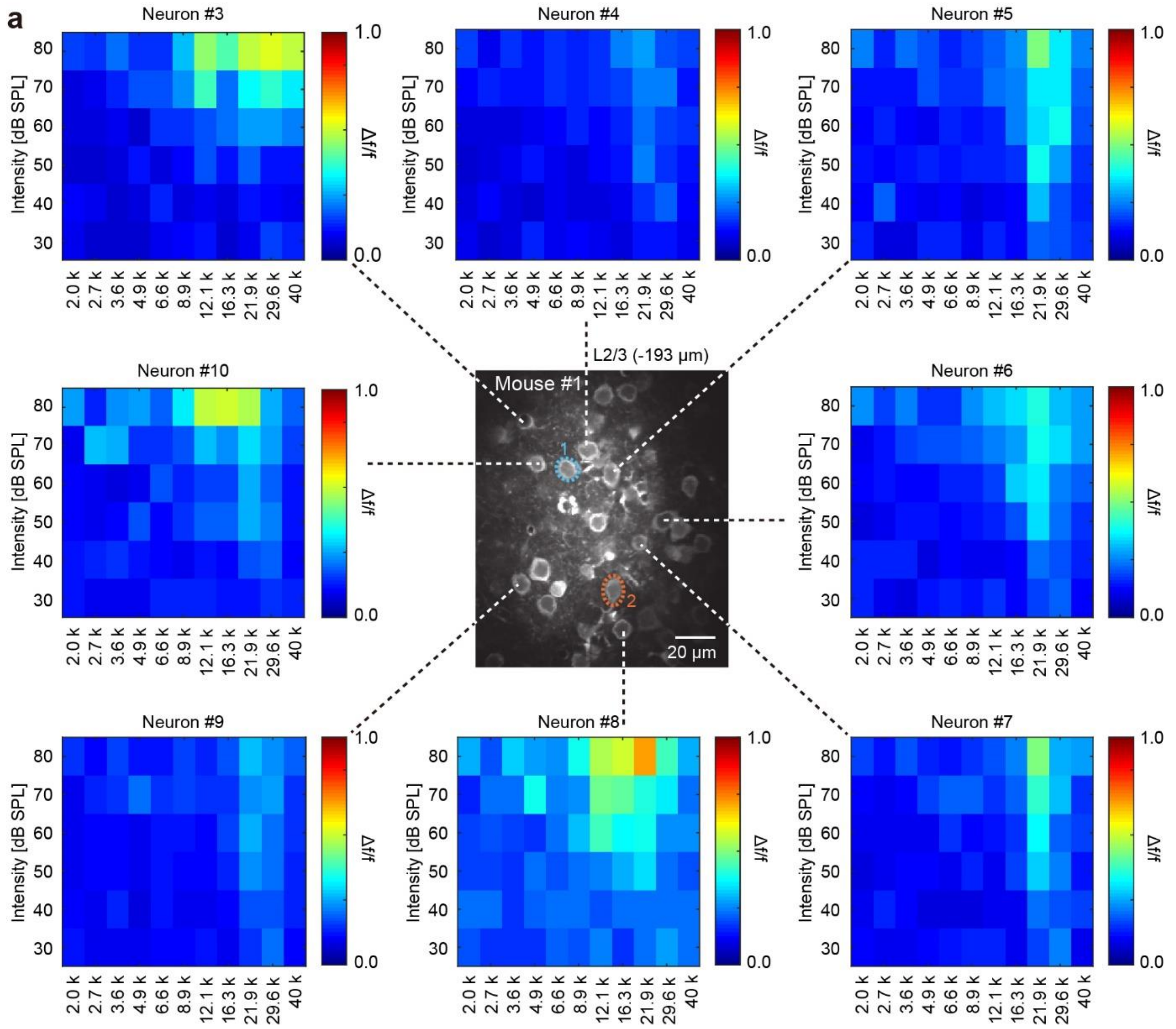

Supplementary Fig. 1 FRAs of individual neurons in L2/3 of the AUD recorded by two-photon Ca^{2+} imaging. Centre panel: Representative two-photon image in L2/3 of the AUD (depth is 193 μm from the pial surface). Surrounding panels: Colour-coded FRAs of individual neurons in the imaging plane. The dashed lines indicate the soma location of each neuron.

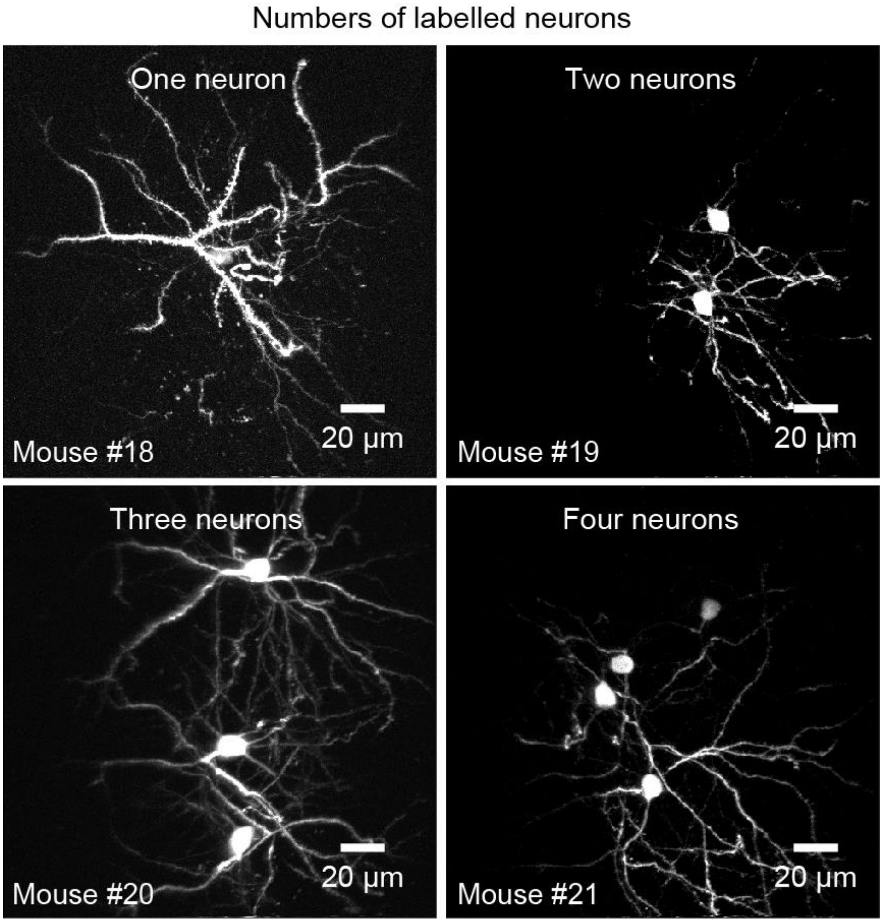

Supplementary Fig. 2 Controlled labelling of 1-4 neurons within a field of view by electroporation.

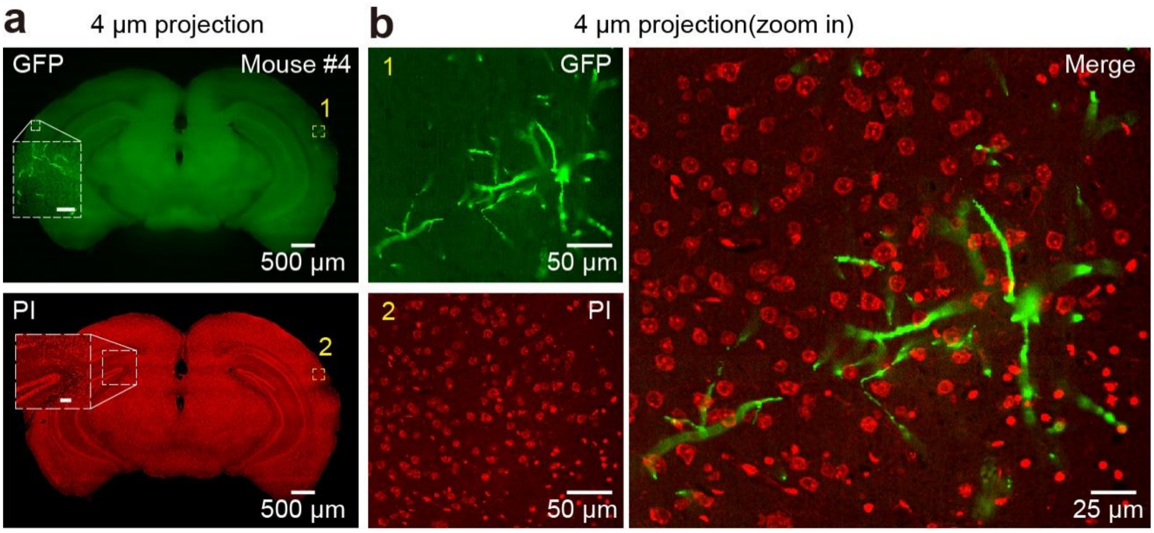

Supplementary Fig. 3 Whole-brain dual-colour imaging. **a** Maximum intensity projections of two serial coronal sections in the GFP channel (upper) and PI channel (lower). **b** Enlarged views of the areas outlined by the yellow dashed boxes in **a**. Left: two channels shown separately; right: merged image.

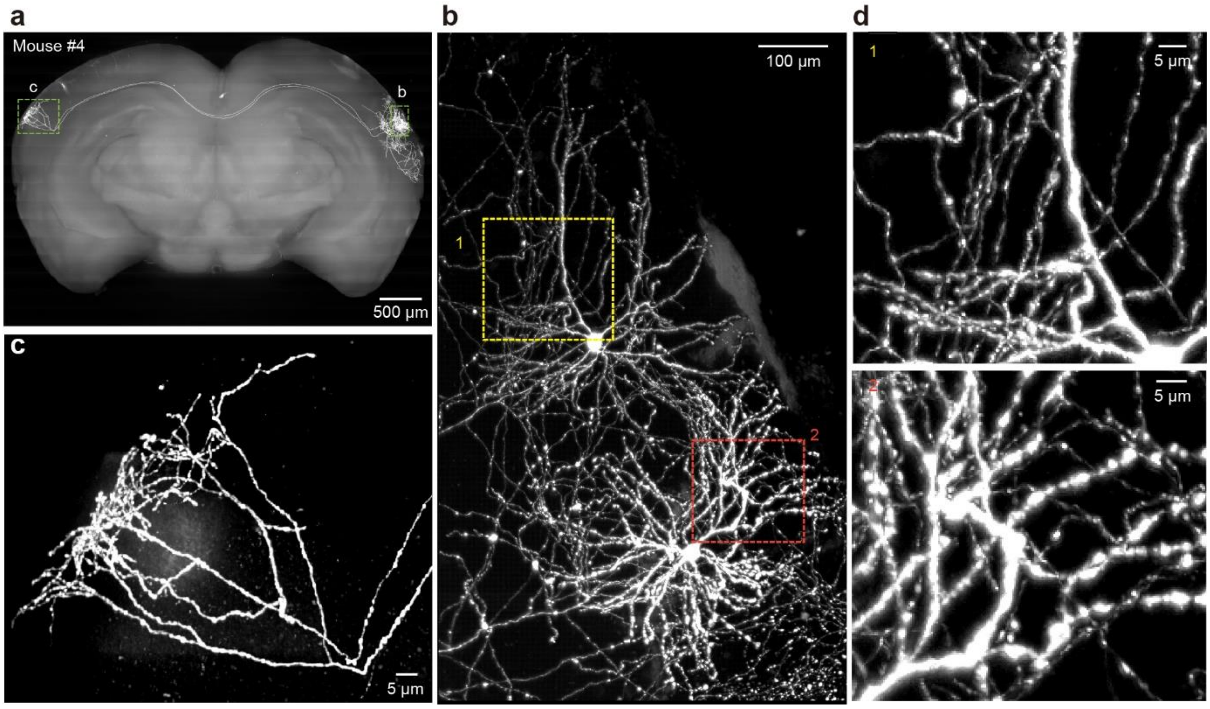

Supplementary Fig. 4 Representative images of whole-brain raw signals of labelled neurons in the AUD. **a** Coronal view of whole-brain maximum intensity projections. **b-c** Enlargement of the corresponding boxes in **a** showing labelled neurons in the injection site (**b**) and contralateral axonal arborizations (**c**). **d** Enlargement of the respective boxes in **b** showing the fine structures of dendrites.

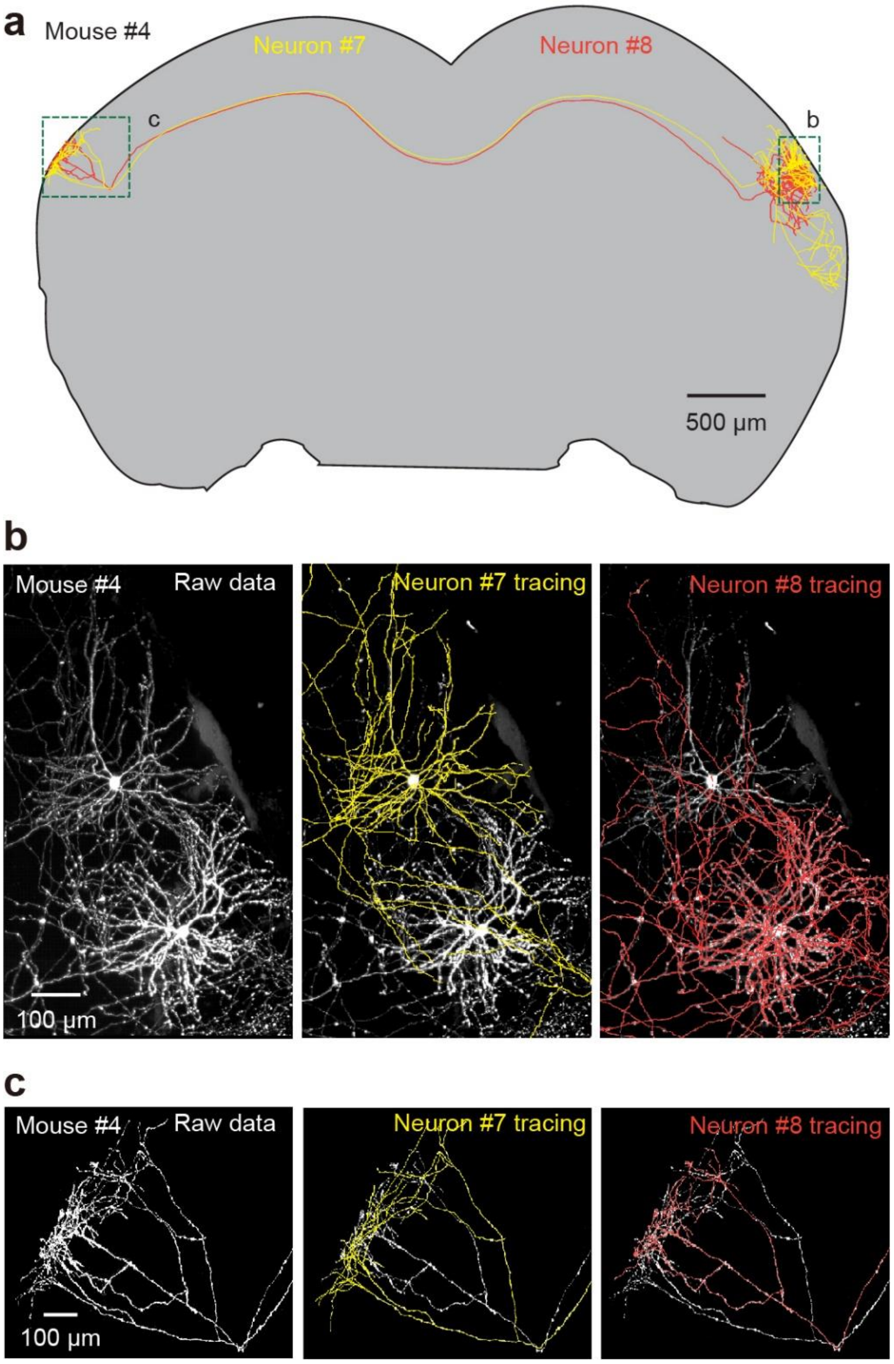

Supplementary Fig. 5 Complete manual reconstruction of individual neurons. a Reconstruction of two representative neurons with long-range projections. Each colour indicates one neuron. **b** Left: image stack of neurites and somata of two reconstructed neurons. Middle to right: reconstructed dendrites of neurons #7 and #8, respectively. **c** Left: image stack of axonal arborizations of the two reconstructed neurons. Middle to right: reconstructed terminal structures of neurons #7 and #8, respectively.

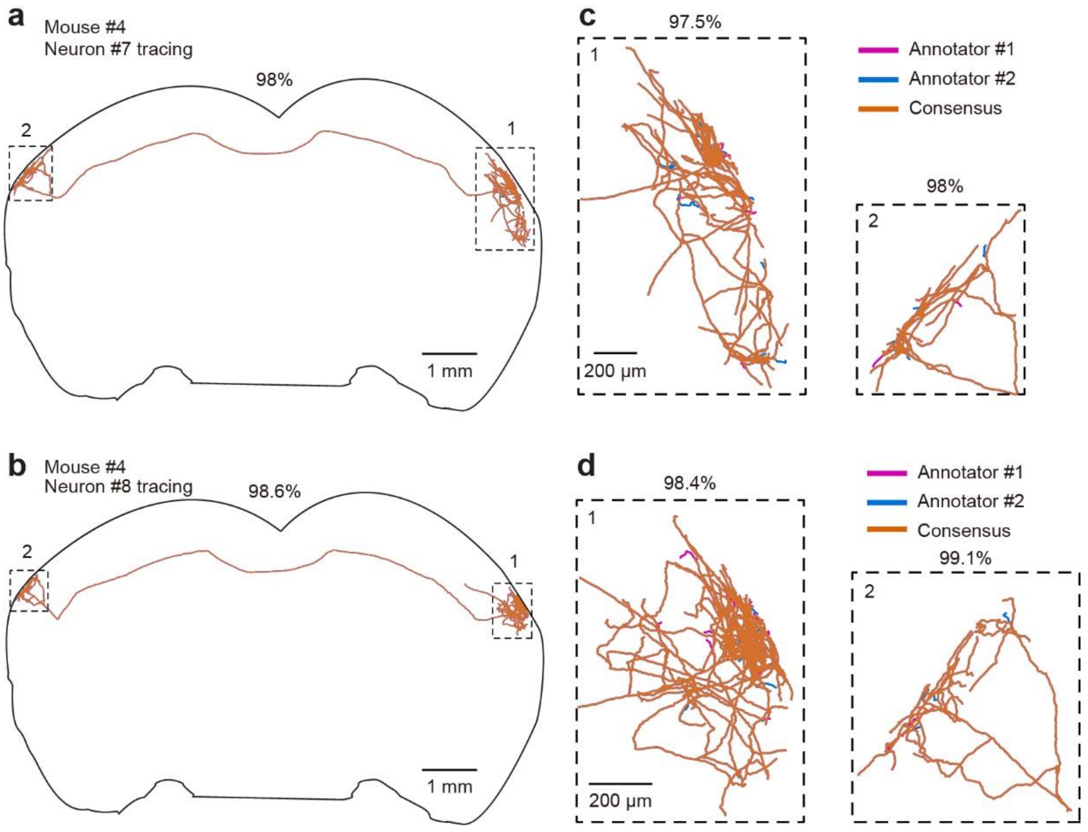

Supplementary Fig. 6 Validation of the accuracy of manual tracing. a-b Overview of two long-range projecting neurons (neurons #7 and #8) reconstructed by two experienced annotators. **c-d** Enlargement of the respective boxes in **a** and **b** showing the details of tracing by annotator #1 (pink) and annotator #2 (cyan) and the consensus between the two annotators (orange).

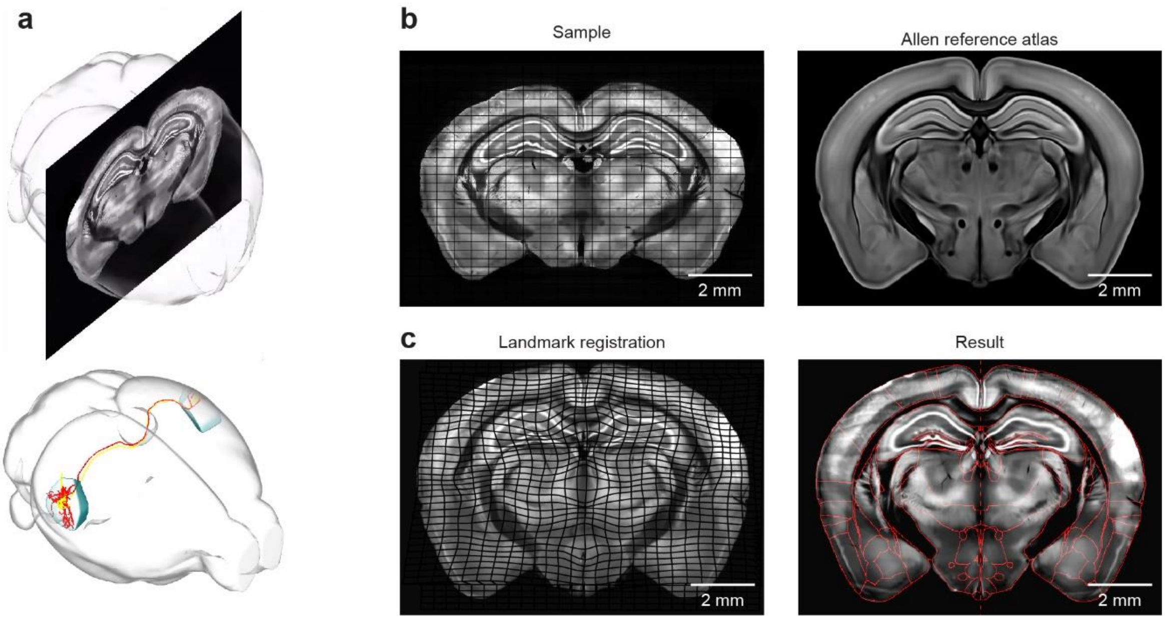

Supplementary Fig. 7 Representative images of reconstructed neurons registered to the standard Allen Brain Atlas. a Raw brain image with overlaid neurites aligned to the template of the Allen Brain Atlas. **b-c** A representative coronal section selected from **a** (**b**; left) was registered to the template of the Allen Brain Atlas (**b**; right) after dense landmark-based 2D registration (**c**; left) with the local-regions approach (**c**; right).

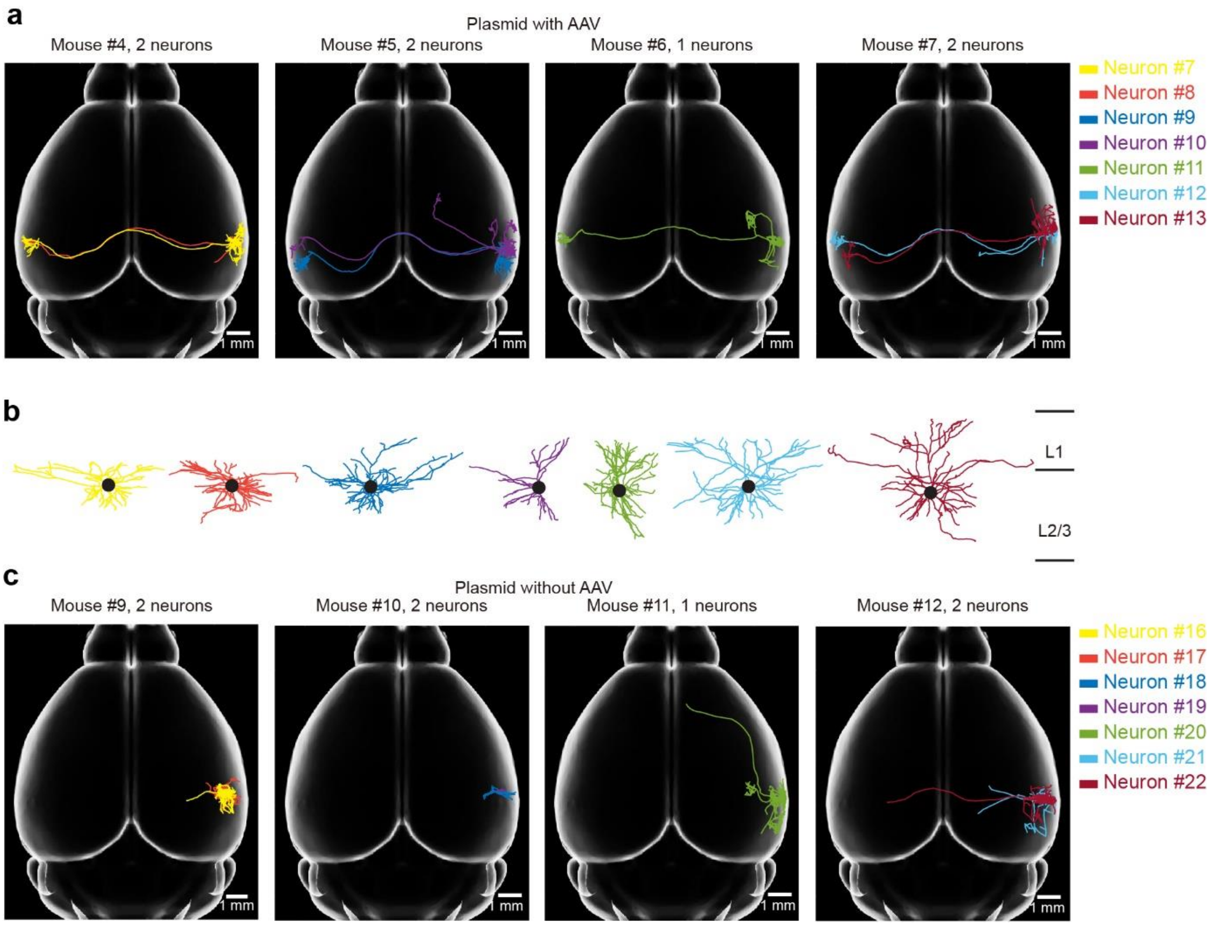

Supplementary Fig. 8 Comparison of the reconstructed neurons labelled with different methods. a Horizontal view of the reconstructed individual neurons labelled by plasmid with local AAV injection. The results for four mice are shown separately. **b** Enlarged view of the dendrites of the reconstructed neurons shown in **a**. **c** Horizontal view of the reconstructed individual neurons labelled by plasmid without AAV injection. The results for four mice are shown separately.

Supplementary Table 1 Troubleshooting guide.

Step	Critical milestone	Problem	Possible reason	Solution
Step 1: Functional identification of neurons by 2P Ca^{2+} imaging [1-5]	1, Perform surgery for 2P imaging (be very careful not to damage the cortex and keep the dura as intact as possible) *	Brain damage	Inaccurate thinning or improper pressing onto the skull	Thinning as gently as possible and do not press the skull
	2, Perform bolus loading of a Ca^{2+} dye (the standard Ca^{2+}-free Ringer's solution is used to minimize precipitation of the dye) *	Cells are not stained	The staining electrode gets clogged or the tissue is damaged	Monitor the pipette resistance during dye injection
	3, Wait for an hour to obtain a stable maximal fluorescence level for stained neurons	The stained dim cells emit too little fluorescence to be detected in vivo	The waiting time is either too long or too short	A stable level of fluorescence within stained cells was reached 1 h after dye injection, from which recording was started and continuous experiments were achievable with duration of 4-6 h
	4, Perform Ca^{2+} imaging and sound stimulation (monitor photobleaching and adjust excitation power to levels just below the bleaching threshold)	Ca^{2+} signals are absent in neuronal somata over multiple trials	Dye bleaching or photodamage occurs due to high laser power during recording	Reduce the laser power as much as possible
Step 2: Targeted labelling of functionally identified neurons [6, 7]	1, Electroporate a plasmid to a targeted neuron (no clogging is critical) ***	Low success rates	1, pipette clogged; 2, inaccurate distance between the pipette and the neuron; 3, sub-optimal electroporation parameters	1, change a new pipette; 2, target the center of the cell soma before electroporation; 3, optimize the amplitude and/or number of voltage pulses during electroporation
	2, Inject AAV locally (distance from the injection site to the electroporated neuron is critical) ***	Weak and uneven fluorescence signal intensity in our electroporated neurons	No enough virus particles taken up	The distance between the virus injection site and the electroporated neuron is within the range of 50 μm and 300 μm
	3, Cranial window implantation (chronic imaging in awake mice over several weeks is critical) ***	Chromic imaging is no longer possible in the weeks following surgery	1, coverglass is dirty; 2, insufficient application of vetbond and/or dental acrylic; 3, inflammatory reaction or skull regrowth	1, clean coverglass with 70% ethanol before application; 2, Make sure all the edges and exposed skull are covered with dental acrylic; 3, treat the mouse with carprofen, sulfamethoxazole + trimethoprim for 7–10 d and try imaging again after 7–10 d
Step 3: Brain-wide dual-colour imaging [8]	1, Whole mouse brain preparation (fixation in PFA and rinse in PBS are critical)	1, brain with strong spontaneous fluorescence; 2, morphological changes in neurons in the brain tissue	1, too much blood residue in the brain; 2, mouse is too young or perfusion rate is too high	1, prolong the time of PBS perfusion; 2, use an adult mouse or reduce the rate of perfusion
	2, Embedding (sample polymerization are critical) *	Morphological changes in neurons in the brain tissue	Initial polymerization temperature is too high	Set initial polymerization temperature to 50°C
	3, Perform fMOST imaging (continuous whole-brain imaging of green and red channels simultaneously is critical)	Incomplete or too large whole-brain data	Improper imaging parameters are set	Set the imaging parameters, e.g., range of interest and exposure time, properly
Step 4: Single-cell reconstruction and analysis [9, 10]	1, Reconstruct complete morphology of individual neurons by manual (two annotators reaching consensus for reconstructions is critical) *	Inaccurate and incomplete reconstructions of individual neurons	Poor signal-to-noise ratio or from occasional attentional drift of individual annotators	Reconstruct neurons with consistent fluorescent signal throughout the entire axonal arbor and every neuron is reconstructed by two annotators
	2, Perform image registration and quantitative analysis at whole-brain level (accurate registration for whole-brain datasets is critical) *	Inaccurate transformation of brain structures in three dimensions	No sufficient feature points are extracted accurately to ensure the registration quality	All the reconstructed neurons are registered to the template of the Allen Brain Atlas by combining the approaches of greyscale-based 3D registration and dense-landmark-based 2D registration in the local regions

References

[1] Chen, X. et al. Functional mapping of single spines in cortical neurons in vivo. Nature 475, 501-505 (2011).

[2] Rothschild, G., I. Nelken , and A. Mizrahi . Functional organization and population dynamics in the mouse primary auditory cortex. Nature Neuroscience 13, 353-360 (2010).

[3] Tischbirek, C. H. et al. In Vivo Functional Mapping of a Cortical Column at Single-Neuron Resolution. Cell Reports 27, 1319-1326 (2019).

[4] Stosiek, C. et al. In vivo two-photon calcium imaging of neuronal networks. Proceedings of the National Academy of Sciences of the United States of America 100, 7319-7319 (2003).

[5] Olga, et al. Targeted bulk-loading of fluorescent indicators for two-photon brain imaging in vivo. Nature protocols 1, 380-386 (2006).

[6] Kitamura, K. et al. Targeted patch-clamp recordings and single-cell electroporation of unlabeled neurons in vivo. Nature Methods 5, 61-67(2008).

[7] Judkewitz, B. et al. Targeted single-cell electroporation of mammalian neurons in vivo. Nature Protocols 4, 862-869 (2009).

[8] Gong, H. et al. High-throughput dual-colour precision imaging for brain-wide connectome with cytoarchitectonic landmarks at the cellular level. Nature Communications 7, 12142 (2016).

[9] Winnubst, J. et al. Reconstruction of 1,000 Projection Neurons Reveals New Cell Types and Organization of Long-Range Connectivity in the Mouse Brain. Cell 179, 268-281 (2019).

[10] Ni, H. et al. A Robust Image Registration Interface for Large Volume Brain Atlas. Scientific Reports 10, 2139 (2020).

* The asterisk indicates the step is critical. The number of asterisks indicates the critical degree of the step.

Supplementary Table 2 Numbers of labelled cells after electroporation and labelling success rate in consecutive experiments.

Labelling success rate					
Mouse number	1h after electroporation	spontaneous activity	5 day after electroporation	10 day after electroporation	30 day after electroporation
Mouse #1	2	2	2	2	2
Mouse #2	3	3	2	2	2
Mouse #3	2	2	2	2	2
Mouse #4	2	2	2	2	2
Mouse #5	4	2	2	2	2
Mouse #6	2	2	1	1	1
Mouse #7	2	2	2	2	2
Mouse #8	2	2	2	2	2
Mouse #9	2	2	2	2	2
Mouse #10	2	2	2	2	2
Mouse #11	2	2	1	1	1
Mouse #12	4	3	2	2	2
Mouse #18	1	1	1	1	1
Mouse #19	2	2	2	2	2
Mouse #20	3	3	3	3	3
Mouse #21	4	4	4	4	4
Total neuron numbers	39	36	32	32	32
Success rate		92%	82%	82%	82%

Supplementary Table 3 Morphological parameters of the reconstructed neurons labelled with different methods.

Our method (Plasmid+virus） in auditory cortex							
Mouse number	Neuron number	Soma location	Dendrite			Axon	
			Length (μm)	Dendrite number	Branch number	Length (μm)	Branch number
Mouse #1	Neuron #1	AUDp2/3	5,896	9	121	74,056	543
	Neuron #2	AUDp2/3	2,321	6	50	25,045	161
Mouse #2	Neuron #3	AUDp2/3	4,830	7	125	41,656	241
	Neuron #4	AUDp2/3	4,135	7	95	28,969	163
Mouse #3	Neuron #5	AUDp2/3	5,075	10	128	61,375	620
	Neuron #6	AUDp2/3	4,498	7	123	52,658	549
Mouse #4	Neuron #7	AUDp2/3	3,212	7	111	46,409	265
	Neuron #8	AUDp2/3	4,238	10	126	40,331	280
Mouse #5	Neuron #9	AUDp2/3	4,137	8	128	43,030	329
	Neuron #10	AUDp2/3	2,029	6	58	60,538	517
Mouse #6	Neuron #11	AUDp2/3	5,766	7	135	45,071	322
Mouse #7	Neuron #12	AUDp2/3	6,674	6	127	40,003	163
	Neuron #13	AUDp2/3	6,184	7	106	43,149	199
Only plasmid in auditory cortex							
Mouse #8	Neuron #14	AUDp2/3	4,044	8	88	27,851	153
	Neuron #15	AUDp2/3	3,702	9	102	45,345	237
Mouse #9	Neuron #16	AUDp2/3	4,235	8	104	34,650	277
	Neuron #17	AUDp2/3	4,193	8	110	35,786	251
Mouse #10	Neuron #18	AUDp2/3	2,362	8	89	14,264	91
	Neuron #19	AUDp2/3	3,358	8	118	521	11
Mouse #11	Neuron #20	AUDp2/3	5,176	7	137	44,492	336
Mouse #12	Neuron #21	AUDp2/3	5,190	8	106	41,931	203
	Neuron #22	AUDp2/3	5,680	10	111	46,295	237
Sparse labelling system (only virus) in auditory cortex							
Mouse number	Neuron number	Soma location	Dendrite			Axon	
			Length (μm)	Dendrite number	Branch number	Length (μm)	Branch number
Mouse #13	Neuron #23	AUDp2/3	6,581	8	162	41,416	269
Mouse #14	Neuron #24	AUDp2/3	4,608	9	96	54,782	327
Mouse #15	Neuron #25	AUDp2/3	4,530	7	119	49,379	343
	Neuron #26	AUDp2/3	4,242	8	104	30,238	167
	Neuron #27	AUDp2/3	4,861	6	97	35,163	179
	Neuron #28	AUDp2/3	3,047	5	61	29,883	129
Our method (Plasmid+virus） in motor cortex							
Mouse number	Neuron number	Soma location	Dendrite			Axon	
			Length (μm)	Dendrite number	Branch number	Length (μm)	Branch number
Mouse #16	Neuron #29	MO2/3	6,603	7	129	47,559	460
	Neuron #30	MO2/3	5,825	9	88	55,917	301
	Neuron #31	MO2/3	4,113	4	58	27,629	147
	Neuron #32	MO2/3	6,334	9	98	48,251	197
Mouse #17	Neuron #33	MO5	6,189	7	95	96,726	517
	Neuron #34	MO5	9,481	11	118	211,821	773
	Neuron #35	MO5	7,939	12	98	109,834	499
	Neuron #36	MO5	7,432	17	93	77,114	363

Supplementary Table 4 Target areas of the reconstructed neurons in the auditory cortex.

The target areas of neurons in the auditory cortex																				
Mouse	Neuron	Isocortex																STR	fiber tracts	axon total
		AI_i	AUD_i	AUD_c	MO_I	ssp_i	SSP_c	SSs_i	SSs_c	TEa_i	TEa_c	VIS_i	VIS_c	VISC_i	LA_i	GU_i	ECT_i	CP_i		
#1	#1		41232	13361				360		856	349							5708	12190	74056
	#2		8768							510					105			14606	1056	25045
#2	#3		25588	6610					629	1161									7668	41656
	#4		12328	6695						118									9828	28969
#3	#5	515	22388	10378		353		385	818	668	536	1034		731	441			12969	10674	61375
	#6	561	16866	5051	3825	4771	3736	3938	3147	874		3106							7344	52658
#4	#7		25811	10735						555									9308	46409
	#8		23678	7547						223	111								8772	40331
#5	#9		15461	1809						8103	6975								10682	43030
	#10	366	38162	8426		1862				1644		1694			358		915		7477	60538
#6	#11		28390	3002						914	427	1625	704						10009	45071
#7	#12		15104	10236						1211		3426							10026	40003
	#13	156	19578	3955				5450		1495	158		587	1467			408		10051	43149
#13	#23	627	34013					231		1063				2866	177	623	113	171	2159	41416
#14	#24		21981	13860					120	1661	193		587					5100	11280	54782
#15	#25		37257					2594										194	9334	49379
	#26		16016					419		1998	126		1103						10576	30238
	#27		14233	12574						184									8172	35163

Supplementary Table 5 Target areas of the reconstructed neurons in the motor cortex.

The target areas of neurons in the motor cortex										
Mouse	Neuron	Isocortex_i	Isocortex_c	MO_i	MO_c	STR_i	STR_c	fiber tracts_i	fiber tracts_c	axon total
#16	#29	7324	0	32178	0	7212	0	845	0	47559
	#30	17273	0	25482	9458	0	0	1692	2012	55917
	#31	0	0	7593	16627	0	0	2044	1365	27629
	#32	16650	1410	17542	3026	6141	0	2055	1427	48251
#17	#33	2386	825	41831	15568	14826	15546	3128	2616	96726
	#34	35968	20083	26350	27432	44342	47852	3819	5975	211821
	#35	12037	10575	16144	18344	37186	7912	4286	3350	109834
	#36	13731	925	14330	26401	14708	2228	2573	2218	77114
